# Emotion‐Focused Therapy: A Perspective From Traditional Chinese Medicine

**DOI:** 10.1002/jhbs.70057

**Published:** 2026-06-15

**Authors:** Wan‐chi Wong

**Affiliations:** ^1^ Department of Educational Psychology The Chinese University of Hong Kong Hong Kong China

**Keywords:** Emotion‐focused therapy, process model of emotion regulation, rational emotive behavior therapy, Spinoza's *Ethics*, traditional Chinese medicine

## Abstract

In clarifying that Spinoza is not the first person to share insights about the necessity of a contrary, strong emotion in transforming an emotion, the present article focuses on expounding this perspective as embedded in the classical texts of Chinese medicine, along with illustrative examples of emotion‐focused therapy in the historical case records of Chinese master physicians. Key features of this kind of emotion‐focused therapy include (1) designing a new scenario that provides the client with an embodied alternate experience in the real world, thereby eliciting a strong emotion capable of overcoming the previously indulged one, and (2) implementing the scenario through collaboration with a person close to the client, or by securing the client's full trust in the physician. A comparison between the role of emotions in the holistic system of traditional Chinese medicine and the philosophical system of Spinoza, as presented in *Ethics*, reveals more variability than commonality. Even though the role of emotion in human functioning is recognized in Spinoza's system of thought, his approach to emotion regulation is fundamentally rationalistic. A closer review of contemporary psychological science and psychotherapy further reveals that cognition plays an intriguing role in the regulation and transformation of emotions. Against this background, the contributions and limitations of emotion‐focused therapy in traditional Chinese medicine are evaluated, followed by the elucidation of its principles and the consideration of its integration with advancements in contemporary psychotherapy.

## 1 Introduction

The role of emotion and its regulation in human life is increasingly recognized by psychologists (e.g., DeSteno et al. 2013; Gross 1998, 2015a). Among the diversified types of psychotherapies, emotion‐focused therapy has emerged as a coherent approach highlighting the awareness, regulation, and transformation of emotions (see Greenberg 2004, 2012, 2002/2015, 2011/2017; Pos and Greenberg 2007). In this field, Spinoza is credited as the first person to note that “emotion is needed to change emotion” (Greenberg 2004, 10). This view is based on a proposition made by Spinoza in his *Ethics*: “An emotion cannot be restrained or taken away except through an emotion that is contrary to and stronger than the emotion that is to be restrained” (Spinoza, 1677/2018, Proposition 7, Part IV, 165). Nonetheless, Spinoza is not a pioneer in sharing this kind of insight. This perspective was clearly explicated much earlier in the classics of Chinese medicine. Interesting examples of emotion‐focused therapy have been found in the therapeutic case records of Chinese master physicians. This article aims to expound the origin and key features of emotion‐focused therapy in traditional Chinese medicine. The commonality and variability between the holistic system of traditional Chinese medicine and the philosophical system of Spinoza, deliberated in an excursion, shed new light on the contemporary scene of psychotherapy. The contributions and limitations of emotion‐focused therapy in traditional Chinese medicine are then discussed in the context of contemporary advancements in psychotherapy.

## 2 On the Emotion‐Focused Therapy of Traditional Chinese Medicine

Concerning traditional Chinese medicine, its emphasis on the holistic relationship between the natural environment and the human body is well‐known in both Chinese and Western cultures (see Kong 2010). Contrasting with its negligible role in the dominant discourses of Chinese philosophy, emotion plays a visible role in the physical and mental health discourses of Chinese medicine. This is most clearly demonstrated in classical texts compiled in *Suwen* 素問 [*Basic Questions*] and *Lingshu* 靈樞 [*Divine Pivot*] of the *Huangdi neijing* 黃帝內經 [*The Yellow Emperor's Inner Canon*] (see Ren 1986), as well as in numerous therapeutic case records from master physicians (see, for instance, Peng 1998; Z. Yu 1709–1799/2008; B. Zhang 1996). Essentially, an excess of emotion, both positive and negative, was regarded as harmful to physical health. Case reports, entitled *yi'an* 醫案, were produced by Chinese physicians from different generations. Among other therapeutic approaches, case reports documented how physicians used emotion‐focused therapy to transform the dominant emotion of their clients. Traditional Chinese medicine's discourses on the role of emotions in the maintenance of health, along with its practice of emotion‐focused therapy, are valuable cultural resources that deserve closer examination.

### 2.1 Discourses on Emotions in the Classics of Chinese Medicine

Impactful discourses on emotions in Chinese culture can be traced back to *The Yellow Emperor's Inner Canon*, the source of wisdom in the theory and practice of Chinese medicine. Its earliest contributors may have been archive clerks who served at the imperial court prior to the Qin dynasty (221–206 BCE), even though its compilation was probably completed in the Han dynasty (206 BCE–220) (Kong 2010, xxxiii). The texts, which were compiled into two volumes called *Basic Questions* and *Divine Pivot*, each containing 81 chapters, have also been continuously re‐edited and rearranged throughout their history (see Ren 1986; Su 2022). The legend of their being dialogues between a respectable ancient emperor and his ministers, such as Qi Bo 歧伯 and Gui Yuqu 鬼臾區, helped to initially establish the authority of the texts. Generations of Chinese physicians, engaging in the thorough study and interpretation of *The Yellow Emperor's Inner Canon*, further enhanced its status as a classic.

The role of emotional regulation in the maintenance of health was highlighted in *The Yellow Emperor's Inner Canon* with an underlying perspective of a holistic interaction between nature and the human body.^1^ How an excess of emotion could harm the body and how that harm could be remedied were crystallized by the following phrases in two influential texts incorporated in *Basic Questions*:^2^

怒傷肝、悲勝怒 Anger hurts the liver; sadness overcomes anger.

喜傷心、恐勝喜 Joy hurts the heart; fear overcomes joy.

思傷脾、怒勝思 Deliberation hurts the spleen; anger overcomes deliberation.

憂傷肺、喜勝憂 Worry hurts the lung; joy overcomes worry.

恐傷腎、思勝恐 Fear hurts the kidney; deliberation overcomes fear.

*Annotations on Basic Questions of The Yellow Emperor's Inner Canon* (1982/1995), vol. 1, 62–97 & vol. 2, 860–890.

The above statements emphasize how (excessive) emotions could hurt corresponding human organs.^3^ The insight these statements provide into how a strong emotion could be applied to overcome another strong emotion has become a source of inspiration for Chinese physicians looking to develop unique methods of emotion‐focused therapy. These methods of therapy were subsequently reported in *yi'an*, the therapeutic case records.

### 2.2 Therapeutic Case Records and Emotion‐Focused Therapy in Traditional Chinese Medicine

Along with the interpretation of the classical texts in *The Yellow Emperor's Inner Canon*, the drafting of *yi'an* constitutes another set of important discourses in the history of Chinese medicine. Taking their earliest shape prior to the Qin dynasty, these medical case records in their specialized form emerged in the Song dynasty (960–1279). The genre then matured in the Ming dynasty (1368–1644) and prospered in the Qing dynasty (1644–1911) (Tao and Gao 2012). Individual Chinese physicians published their *yi'an,* and their works were further selected and compiled into collected works.^4^ These therapeutic case records are regarded as important because they often constituted an integration of theory and practice in Chinese medicine. The flourishing publication of *yi'an* could probably be attributed to the needs of a new generation of physicians from the scholar‐gentry class, who were eager to consolidate their expertise and reputation through such publishing; in addition, these published case records simultaneously posed a challenge to the authority consolidated by other physicians who had inherited their positions via family tradition (Cullen 2001). Emotion‐focused therapy was only one therapeutic approach among several practiced in Chinese medicine, with herbal treatment and acupuncture being the two dominant approaches. Although occupying a relatively inconspicuous position in the *yi'an* literature, emotion‐focused therapy remained a cherished tradition derived from the insights embedded in *The Yellow Emperor's Inner Canon* and particularly flourished in the Jin (1127–1279), Yuan (1271–1368), Ming (1368–1644), and Qing (1644–1911) dynasties.

In the contemporary literature of Chinese medicine, emotion‐focused therapy is called *qingzhi xiangsheng liaofa* 情志相勝療法, “the therapy of overcoming an emotion with another emotion.” This therapeutic approach has been briefly introduced in the field of Chinese medical psychology and in its subfield of psychotherapy in Chinese medicine, both of which emerged in China in the 1980s and continue to develop into the 21st century, although the psychological perspective was embedded early on in the classical texts and practices of Chinese medicine (see Dong 2015; Hao 2009; M. Wang 1985; B. Zhang 1996; Zhuang 2007/2013, 2015). The first case of using emotion‐focused therapy is often traced back to a story of how the master physician Wen Zhi 文摯 cured Emperor Qi by consciously arousing his anger in multiple ways, recorded in *Master Lu's Spring and Autumn Annals* (Lu 292–235 BCE/2011, 315–317, see also M. Wang 1988). The application of emotion‐focused therapy became active and well‐recorded in the Jin, Yuan, Ming, and Qing dynasties. Zhang Zihe 張子和 (1156–1228), a distinguished physician of the Jin dynasty, gave impetus to this approach by designing the therapy in a creative way (see Z. Zhang 1156–1228/2017). Wu Kun 吳崑 of the Ming dynasty further articulated that a sickness caused by an overwhelming emotion cannot be cured by medicine but should be overcome by another emotion (K. Wu 1551–1620/2007, 146). It is worth noting that cases of emotion‐focused therapy constitute an independent section entitled *qiqing* 七情, meaning “seven kinds of emotions” in some but not all collected works of *yi'an* (e.g., Peng 1998; Z. Yu 1709–1799/2008). It is further interesting to note that contemporary literature in Chinese medicine continues to illustrate examples of emotion‐focused therapy from the “golden period” of the Jin, Yuan, Ming, and Qing dynasties without supplementing newly designed cases (see, for instance, Peng 1998; Y. Zhang 2007; Zhuang 2015).^5^

The following five case records were selected to illustrate the unique approach of emotion‐focused therapy as conducted by Chinese physicians in the Jin, Yuan, Ming, and Qing dynasties:

1. “Sadness overcomes anger.”
  Subsequent to a quarrel, a person was filled with great anger and appeared to be very sick. Realizing that excessive anger was the cause of the sickness, physician Zhang Jiebin 張介賓 (1563–1642) of the Ming dynasty proposed a special therapeutic approach that would create a great deal of pain, including hurting the face and the skin of other body parts. This proposal first created fear in the patient; he further felt sad when he thought of his doomed future. His anger could thus be released.^6^
2. “Fear overcomes joy.”
  A man who came first in the highest imperial examination and received the title of Zhuangyuan got very sick. Physician Xu Lingtai 徐靈胎 (1693–1771) of the Qing dynasty recorded how he treated this patient. He simply told the patient to return home immediately because he was expected to live only seven days. This patient then headed for home, together with his servant. Physician Xu gave a letter to the servant and instructed him to let his master open it when arriving home. The patient was cured when he arrived home and read the letter. Xu realized that the sickness of the Zhuangyuan was caused by an excess of joy; a strong feeling of fear helped him overcome that excess.^7^
3. “Anger overcomes deliberation.”
  A woman got very sick with excessive deliberation. She could not sleep well for 2 years, and no medicine could help her. When physician Zhang Zihe (1156–1228) of the Jin dynasty was invited to cure her, he worked out a “therapeutic plan” together with her husband. Zhang enjoyed eating and drinking with the husband at the home of the patient for a few days without putting any effort into suggesting a cure. He also took a hefty sum of money away when he left. The wife became extremely angry and sweated, and then fell asleep without waking up for eight to nine days. Then, she was able to eat again and became well.^8^
4. “Joy overcomes worry.”^9^
  Physician Zhu Danxi 朱丹溪 (1281–1358) of the Yuan dynasty was consulted by Zhuangyuan Chen about curing his brother, who worried a great deal and could not recover from coughing and vomiting blood. Zhu suggested that joy should be created to resolve the client's worry. After obtaining a place to settle where his basic needs in life were no longer a concern, the client rejoiced and recovered immediately without taking any medicine.^10^
5. “Deliberation overcomes fear.”
  A woman experienced a theft and fire in an inn and fell out of bed from the shock. She then became very frightened of all kinds of noises for over a year. When her husband consulted physician Zhang Zihe, the physician implemented a therapeutic plan that involved guiding the woman to engage in deliberation about the noises around her. He did this first by making great noises in front of her, followed by noises behind her back and at night. The state of repeated deliberation over these noises thus helped her to overcome the sense of fear.^11^

### 2.3 Music Therapy in Traditional Chinese Medicine

While the treatment of the above cases is basically emotion‐focused, it is operated by designing and setting up a new scenario, through which a strong emotion can be transformed. The insight of this unique approach lies in its deep understanding of the power of strong emotions, so that a solution is found by eliciting one sort of strong emotion in an appropriate way to overcome the originally indulged emotion. An approach involving music therapy in Chinese medicine, which is by nature emotion‐focused, is also noteworthy. In traditional Chinese music, there are five types of modes reflecting different emotional tones: *gong* 宫, *shang* 商, *jue* 角, *zhi* 徵, and *yu* 羽, originally referred to as a pentatonic scale. These five musical tones and their relationship with physical health were mentioned in *The Yellow Emperor's Inner Canon* and later interpreted and elaborated throughout Chinese history up to the present time (see Kou and Wang 2017; Tsai 2015; S. Wu 2014; Y. Zhang 2019). Unlike Western classical music, which covers a wider sound range, traditional Chinese music is usually characterized by one basic tone of emotion in a single piece. This unique style of composition has probably facilitated the application of a unique approach in the music therapy conducted by Chinese physicians. In his *Chinese Medical Psychology*, Zhang Bohua (1996) summarized three principles of traditional music therapy that have been inherited and applied by contemporary Chinese physicians. The first principle lies in using an appropriate emotional tone of music to overcome another type of dominant emotion in a client. The second principle is actualized by providing music with the kind of emotional tone that is lacking in the client's emotional state. The third principle is characterized by an initial approach of matching the client's emotional state with a congruent emotional tone of music, followed by guiding the client to arrive at a desirable emotional state with music of another emotional tone. The aforementioned three principles of music therapy could be applied flexibly depending on the needs of clients (B. Zhang 1996, 248–249). There have been divided views on the use of music therapy in traditional Chinese medicine. According to Dong (2015), there is a lack of systemic development of music therapy at both the theoretical and operational levels in traditional Chinese medicine; its application is also not widespread.

### 2.4 The Unique Features of Emotion‐Focused Therapy in Traditional Chinese Medicine

From the aforementioned discourses and practices of emotion‐focused therapy in traditional Chinese medicine, we can clearly identify two core insights: (1) recognition of the harm caused by excessively strong emotions; (2) understanding of the need for a strong, contrasting emotion to overcome the excessively strong emotion.

Anchoring on these basic ideas, Chinese physicians developed practical approaches in their emotion‑focused therapy, as exemplified in therapeutic case records. This practice carries a dominant feature of creating a new scenario in which the excessive emotion could be transformed. While creating a favorable new living condition is understandably helpful in facilitating a positive emotion, as in the case of “Joy overcomes worry,” it is also feasible to create a new scenario mainly through simple narratives, such as the cases of “Sadness overcomes anger” and “Fear overcomes joy.” In the case of “Anger overcomes deliberation,” the client was led to witness a brief but dramatic interaction between the physician and her husband at home. In the case of “Deliberation overcomes fear,” the client was engaged in systematically planned activities in the home setting. The range of the illustrated cases demonstrates the shrewdness and flexibility of these physicians in creating an appropriate scenario for transforming an excessive emotion with a strong, contrasting emotion, depending on the characteristics of the clients and the emotions involved. Another necessary condition for successfully setting up a new scenario, whether simple or complex, was collaboration with a close family member of the client or the client's complete trust in the physician.

The practice of music therapy among Chinese physicians also shares the core insights of the scenario‐creating approach. Building on the unique characteristics of Chinese music, physicians approached music therapy in a shrewd and flexible way, as shown by the principles observed. A piece of music with a strong emotional tone can be used to transform an excessive emotional state in some clients while supplementing the lack of such an emotional state in others. In full recognition of the difficulty in transforming a strong emotion, certain musical pieces would be selected in the initial step to match the emotional state of a client. This would be followed by guiding the client to arrive at the targeted emotional state through further musical pieces of a contrasting emotional tone.

It is worth noting that no explicit record has been found of a debriefing process in the emotion‐focused therapy of traditional Chinese medicine. The preparation of the letter by physician Xu Lingtai in the illustrated case of “Fear overcomes joy” can be perceived as performing a debriefing function. In other cases, the physician might or might not have conducted a debriefing. The highlight of the therapeutic case records lies in the analysis of emotional states, the design of a new scenario, and the effectiveness of the emotional transformation. In both the scenario‐creating approach and the music‐therapeutic approach, it appears that debriefing was not built in as an essential component of the emotion‐focused therapy, or at least was not explicitly stated as one. Given this feature, we are not sure to what extent the clients would have attained the insights about why they were cured effectively, and through this, whether they gained a deeper understanding of their underlying emotional states.

## 3 The Holistic System of Traditional Chinese Medicine and the Philosophical System of Spinoza, as Exemplified in *Ethics*: Exploring Their Commonality and Variability, With Special Reference to the Role of Emotions

It is interesting to observe that a similar insight concerning the important role of emotions in changing emotions is shared in traditional Chinese medicine and the philosophy of Spinoza. This prompts an intriguing exploration into the contexts encapsulating this insight, with the aim of uncovering the commonalities and variations between these two systems of thought.

### 3.1 The Holistic System of Traditional Chinese Medicine

Spinoza's *Ethics* appeared in 1677. By contrast, the Chinese classical texts known collectively as *The Yellow Emperor's Inner Canon*—including *Basic Questions* and *Divine Pivot*—were circulated much earlier, although the legendary framing of the work as a dialogue between an ancient emperor and his ministers is not considered reliable history. The compilation of this theoretical system of Chinese medicine is generally thought to have started during the Warring States period (475–221 BCE) and continued through the Western Han (206 BCE–8 CE) and Eastern Han (25–220) dynasties (Sun 2010). The title *The Yellow Emperor's Inner Canon*, comprising 18 volumes, was first recorded in Ban Gu's catalog in vol. 30 of *Han History* (Ban 32–92/1983, 225), itself based on an earlier compilation by Liu Xin (50 BCE–23 CE). As an individual book, *Basic Questions* was first mentioned in the work of Zhang Ji, a physician of the Eastern Han dynasty. Its contemporary form was shaped and annotated in the Tang period (618–907) by Wang Bing, whose edition contained 24 volumes and 81 chapters (see *Annotations on Basic Questions of The Yellow Emperor's Inner Canon* [1982/1995]). *Divine Pivot* was organized into 24 volumes in a publication by Shi Song in the Southern Song period (1127–1279), while its contemporary version was chiefly established through the major annotation of Ma Shi in the Ming period (1368–1644) (see *Divine Pivot of The Yellow Emperor's Inner Canon: Three Annotation Traditions* [Y. Wang 2013]). During the Ming period, *Basic Questions* and *Divine Pivot* were further reorganized into thematic sections and discussed in two influential works: Zhang Jiebin's *Leijing* (1563–1642/1995) and Li Zhongzi's *Huangdi Neijing: A Synopsis with Commentaries* (see Kong 2010).

Throughout its long history of compilation, annotation, and interpretation, the red thread of this theoretical system in traditional Chinese medicine remains the same. Both the natural environment and the human body have been perceived as a holistic cosmos that operates under the dialectic of two contrasting forces called yin and yang. The continuous and subtle interaction within and between the natural environment and the human body gives impetus to human life, whereas good physical and mental health relies on an understanding of and compliance with nature. In such a theoretical system of dialectical movement, *qi* is postulated as a key construct. As shown in the classical texts of *The Yellow Emperor's Inner Canon*, *qi* carries a number of core features. In the first place, it is the refined, basic substance that constitutes any possible life in the cosmos through its convergence and accumulation. Its existence is by nature a kind of dynamic and fluid movement that is never ceasing. It penetrates the natural environment and gives rise to its order; it also penetrates the human body and makes possible its functioning. Essentially, the continuous interaction of the natural environment and the human body is actualized through the interaction of *qi* (see also L. Zhang, 1990/1994).

What is the role of emotion in the theoretical system of traditional Chinese medicine? While there are no explicit explanations in the classical texts like those in contemporary physiology and psychology, the context of crucial statements (including how a specific emotion hurts a particular organ and how one emotion can be overcome by contrasting with another) was placed within changes in the natural environment.^12^ It is also worth noting that the *qi* of human organs was postulated as conducive to the generation of emotions. Taken together, the genesis of emotions was explained through the interaction of the *qi* in the natural environment and the human body.

How various kinds of emotions hurt the circulation of *qi* was explicitly described in the classical texts. For instance, it was vividly formulated as follows in Chapter 30 of *Basic Questions*:

Huangdi said, “I know all diseases are caused by [disturbance of the righteous] *qi*. The *qi* surges upward when one is angry, slackens when one is joyful, dissipates when one is sad, sinks [is unable to rise] when one is apprehensive, condenses [is unable to disperse] when one is cold, leaks [out of the body] when one is hot, unsettles when one is frightened, depletes when one is tired and entangles when one is pining.”

—“On pain” (translated by Kong 2010, 314).

In other chapters of *The Yellow Emperor's Inner Canon*, an excess of emotions was postulated as being harmful to the *shen* (spirit) of the human body.^13^ The meaning of *shen* in the classical texts of Chinese medicine is manifold and ambiguous, depending greatly on the context in which it appears. As explicated in a standard contemporary textbook, *shen* is generally referred to as carrying the twofold meaning of the governing force of life‐activity and its holistic expression (Sun 2010). Regarding the pathological consequences of excess in emotions, the master physician Zhang Jiebin has made a summary in his masterpiece *Leijing* (J. Zhang 1563–1642/1995: 1121–1125), making faithful use of *The Yellow Emperor's Inner Canon* as his source.

Upon close examination of the classical texts compiled in *The Yellow Emperor's Inner Canon*, it becomes evident that the discourse on emotion does not constitute a prominent or extensively detailed component of the texts. Nonetheless, within the broader context of understanding the natural environment and the human body as a holistic system, an important perspective on emotion and its relationship to well‐being is articulated. In identifying seven essential emotions—referred to as *qíng* (情)—including *xi* 喜 (pleasure), *nu* 怒 (anger), *you* 憂 (worry), *si* 思 (deliberation), *bei* 悲 (sadness), *kong* 恐 (fear), and *jing* 驚 (fright), classical texts in traditional Chinese medicine highlight the harm in regard to health caused by emotional excess and the need to find a remedy in regulation. Notably, master physicians from the Jin, Yuan, Ming, and Qing dynasties gained insights about the important role of a contrasting emotion in transforming a currently dominant one and developed a unique kind of emotion‐focused therapy that is shrewd and flexible, as illustrated in the therapeutic case records. The development of music therapy based on the same insight is also worthy of note.

In seeking to understand the context of traditional Chinese medicine's notion of emotion, engaging in etymological and semantic analyses of emotion words presents itself as a sensible approach.^14^ Three emotion and emotion‐laden words that remain in use today—namely, *xi* 喜 (pleasure), *le* 樂 (joy), and *xin* 心 (heart)—appear in Oracle Bone Script, the oldest Chinese script dating back to the mid‐to‐late Shang dynasty (fourteenth century BCE–1046 BCE). Their original meanings, however, were not related to emotions.^15^ Over time, *xin—*meaning “a human heart”—came to be used as the principal component in creating numerous emotions and emotion‐laden words.^16^ This development reflects the folk concept of relating emotions to the human heart. The emergence of emotion words is witnessed in Bronze Script, a dominant script in the Western Zhou dynasty (1046–771 BCE). Words, including *nu* 怒 (angry), *you* 憂 (worrying), *bei* 悲 (sad), *kong* 恐 (fear), *ju* 懼 (dreadful), *hui* 悔 (regretful), *kui* 愧 (shameful), *yuan* 怨 (animosity), *yang* 怏 (discontented), *yu* 愉 (delightful), and *yi* 怡 (pleasant), were created with *xin* as their principal component,^17^ reflecting a growing awareness and differentiation of emotional states. Notably, a greater number of words were coined in this period to express what we generally consider to be negative emotions. This trend became even more pronounced in Small Seal Script, a standardized script promulgated during the Qin dynasty (221–207 BCE).^18^ *Qing*, an umbrella term for diverse kinds of emotions, was first found in Small Seal Script. This collective term originally referred to an entity that emerged from a person in a natural and authentic way.^19^

Among the seven kinds of emotions identified in the classical texts of traditional Chinese medicine, five words (i.e., *nu* 怒, *you* 憂, *si* 思, *bei* 悲, and *kong* 恐) were created using the principal component of *xin*. As with the newly coined emotion words in Bronze Script and Small Seal Script, the seven emotions highlighted in traditional Chinese medicine are also predominantly negative by nature. Traditional Chinese medicine's perspective on emotions—specifying that an excess of a typically positive emotion can be harmful, while a contrasting negative emotion can serve to counterbalance an excessive emotion—is nevertheless original and unique.

A further endeavor to trace the context of traditional Chinese medicine should be anchored in the *Yijing* 易經 (*The Book of Changes*), which is regarded as the earliest Chinese classic and the root of Chinese cultural thought.^20^ Originating as an oracle book, it evolved from ancient times up to the early Zhou dynasty (ca. 11th century BCE).^21^ According to legend, the Eight Trigrams were created by the cultural hero Fuxi 伏羲, based on his observations of cosmic order and life on earth. Each trigram consists of three lines—either broken or unbroken—symbolizing the contrasting primary forces of yin and yang.^22^ The further construction of the Sixty‐Four Hexagrams was achieved by combining the Eight Trigrams, representing sixty‐four distinct times and situations.^23^ Notably, this system of images was later enriched by textual elements: a judgment for each hexagram and line statements for each of the six lines (broken or unbroken) within a hexagram.^24^ In the Great Treatise (Xici 繫辭), an important commentary in the first round of interpretations of the *Yijing*,^25^ worrying about possible future adversity is portrayed as the impetus behind the creation of the *Yijing*.^26^ Nonetheless, the core message of the *Yijing* does not center on emotion, but rather on the importance of understanding subtle, interactive changes in nature and the human world—particularly in their germinal stages—so that one may respond appropriately and lead an ethical life.^27^ Intriguingly, this perspective may also be interpreted as a way of transcending negative emotions during uncertain times and adverse situations.

The essential role of the dialectic between the contrasting forces of yin and yang in shaping both nature and human existence—serving as the cornerstone of the holistic system of traditional Chinese medicine—finds its roots in the ancient discourses of the *Yijing*. Nonetheless, the postulation of the continuous interaction of *qi* between the natural environment and the human body, along with the articulation of the subtle relationship between *qi*, emotion, and human well‐being, constitutes a coherent and original system of thought within traditional Chinese medicine. This conceptualization of *qi*'s dynamic interplay—together with the recognition of the harm caused by excessive emotions, and the therapeutic use of a contrasting emotion to restore balance—likely originated from the specific concerns and discerning clinical observations of Chinese physicians.

Given the legendary nature of ancient medical and philosophical discourses, along with the complex history surrounding the compilation and interpretation of classical texts, it is often difficult to determine the direction of influence among the various systems of ancient Chinese thought. In full recognition of traditional Chinese medicine as a coherent theoretical system, it is equally important to highlight its openness as a system in connection with the continuous development of Chinese philosophical discourses. While it is evident that Daoist ideas of compliance with nature permeate the classical texts of Chinese medicine, it is also noteworthy that the idea with regard to the necessity of regulating excessive emotions concurs with a central Confucian idea.^28^ However, the Confucian approach to overcoming excessive emotions differs from that of traditional Chinese medicine. Through his well‐known motto, articulated as “*renzhe buyou, zhizhe buhuo, yongzhe buju*” 仁者不憂, 智者不惑, 勇者不懼, Confucius (ca. 551–479 BCE) seems to have suggested that virtues are in the position to transcend negative emotions.^29^ Rituals and music are further presented as means of regulating and cultivating emotions in the *Liji* 禮記 (*The Record of Rites*) and in two key essays by Xunzi 荀子 (ca. 340–245 BCE).^30^ Whether *qi* is the cause of life is not explicitly expounded in *The Yellow Emperor's Inner Canon*. Nonetheless, there has been an extensive discourse on this question in Chinese philosophy. For instance, Liu An, author of the *Huainanzi*—a highly influential philosophical work from the Han dynasty—traced the origin of life to the Dao, rather than to *qi* (see Lau and Ames 1998; Liu 179–122 BCE/1997). Zhang Zai (1020–1077) of the Song dynasty, a key figure in Neo‐Confucianism, conceptualized *qi* as “One,” the source of the cosmos containing all contraries in potential integration. “Two” referred to the contraries themselves, while “Three” signified their synthesis, understood as an enriched “One” (Z. Zhang 1020–1077/1978, 1020–1077/2000). It is also interesting to note that in Confucian and Neo‐Confucian thought, *qi* was often endowed with a moral dimension. This notion reached its height in Zhang Zai's philosophy, where the cosmos was conceived as an extension of the human body and empathetic understanding of the whole and its interactions was cherished as a moral ideal (Z. Zhang 1020–1077/1978, 1020–1077/2000).

### 3.2 The Philosophical System of Spinoza, as Exemplified in *Ethics*

In contemporary psychotherapy, Spinoza was traced back as a pioneer in recognizing the role of emotions in changing emotions (see Greenberg 2004). This is rooted in a proposition made by Spinoza in his masterpiece of philosophy, entitled *Ethics* (Proposition 7 of Part IV; see Spinoza, 1677/2018, 165). This philosophical system is constructed with a geometrical, deductive method of expounding definitions, axioms, propositions, proofs, and corollaries, supplemented by scholia, explanations, and appendices. Its integrated and comprehensive system of thought begins with an inquiry concerning God (Part I), continues through the investigation of the nature and origin of the mind (Part II) as well as the origin and nature of the emotions (Part III), to the examination of human servitude—that is, the strength of human emotions which can hold us in bondage (Part IV). It ends with a discussion of the power of the intellect, which brings us to peace through reason—that is, to human freedom (Part V). The discussion of emotions certainly has an important place in this philosophical system, constituting two out of five parts of the text. It is also clear that Spinoza's dominant concern is how the mind can govern strong emotions (Spinoza, 1677/2018, Preface, Part III, 93–94). How can the proposition about the essential role of emotions in changing emotions be understood in the context of Spinoza's basically rationalistic standpoint? Reconciliation is by no means difficult once we understand Spinoza's postulations on the intimate interconnectedness of body, mind, and emotions, which we will now turn to.

In Part III of *Ethics*, Spinoza expounds on the origin and nature of emotions. He begins with a postulation on the connectedness of body, mind, and emotions. By emotion [*affectus* 31] Spinoza means “affections of the body by which the body's power of action is augmented or diminished, assisted or restrained, and at the same time the ideas of these affections” (Spinoza, 1677/2018, Definition 3, Part III, 95). A human body would be active with adequate ideas and passive with inadequate ideas. Essentially, an emotion is an imagined idea; an emotion in the form of a passion is a confused idea. Spinoza differentiates emotions into three major types: desire, joy, and sadness, under which further kinds of emotions are subsumed. In discussing Spinoza's anatomy of the affects, LeBuffe (2009, 204) has constructed a table illustrating Spinoza's classifications of active affects and passive affects. Active affects include active joy (covering love of God and active self‐esteem) and active desire (covering tenacity, moderation, sobriety, presence of mind, nobility, courtesy, mercy, morality, and being honorable). Passive affects encompass a great variety of passive joy (e.g., pleasure, love, thankfulness, devotion, pride), passive desires (e.g., timidity, ambition, and longing), and passive sadness (e.g., pain, hate, envy, fear, shame). Passive effects are passive in the sense that they are determined by external causes.

In Part IV of *Ethics*, how excessive passions pave the way for human servitude is expounded. Proposition 7 in Part IV is considered to be a pioneering idea regarding the role of emotions in changing emotions by the founder of contemporary emotion‐focused therapy (Greenberg 2004). A closer examination of the Proof and Corollary of Proposition 7 reveals Spinoza's thesis regarding the interconnectedness of the body, emotions, and ideas in the transformation of emotions, which aligns with the definition of emotions at the beginning of Part III in *Ethics*. Spinoza's key argument is encapsulated in the Corollary of Proposition 7 in Part IV, as follows: “Insofar as an emotion is related to the mind, it cannot be restrained or taken away except by the idea of a contrary affection of the body which is stronger than the affection which we are undergoing” (Spinoza, 1677/2018, 165). In relation to this argument, it is worth noting that Spinoza understands contrary emotions as “emotions that draw a person in different directions,” even in cases where they are of the same kind (Spinoza 1677/2018, Definition 5, Preface, Part IV, 160).

In Part V of *Ethics*, it is made clear that the strength of emotions is determined by the power of external causes tested against the power of one's own mind, in which adequate versus inadequate ideas play a crucial role (see Spinoza, 1677/2018, Scholium, Proposition 20, Part V, 235–236).

External causes, adequate versus inadequate ideas, and manifested emotions are manifold, which together constitute a complex whole. How can emotions be governed by the mind? Spinoza suggests that the power of the mind over emotions consists of the following:

First, in cognition of the emotion itself.

Secondly, the fact that it separates the emotions from the thought of an external cause, which we imagine in a confused way.

Thirdly, in the time, by which the affections related to things that we understand surpass those which are related to things that we conceive in a confused or mutilated fashion.

Fourthly, in many causes that foster the affections related to the common properties of things or to God.

Fifthly and finally, in the order in which the mind is able to order and connect its emotions with each other.

—*Ethics* (Spinoza, 1677/2018, Scholium, Proposition 20, Part V, 235).

Whereas imagined ideas constitute the first kind of cognition in Spinoza's scheme of cognition, which are usually inadequate in the sense of being confused and falsehood‐inviting, the above‐mentioned five types of necessarily adequate ideas are conceived as the second kind of cognition, characterized by Spinoza as capable of transcending sense experiences through reasoning upon the foundation of common notions. To attain the ideal of a good life, the third kind of cognition is needed. It proceeds “from an adequate idea of certain attributes of God to an adequate cognition of the essence of things” (Spinoza, 1677/2018, Proof, Proposition 25, Part V, 238). Dialectically, “the more we understand things in this way, the more we understand God” (Spinoza, 1677/2018, Proof, Proposition 25, Part V, 238). In the case that we are delighted in the blessedness of knowing and loving God,^32^ we are able to conquer our passions (see Part V of *Ethics*, Spinoza, 1677/2018). Spinoza understood that this goal is by no means easy to attain. Thus, he closed the last scholium of *Ethics* with the saying that “all noble things are as difficult as they are rare” (Spinoza, 1677/2018, Scholium, Proposition 42, Part V, 250).

Although *Ethics* was composed in a single lifetime, it belongs to a broader Western philosophical conversation, interwoven with continuities and ruptures in ancient, medieval, and modern thought. As Steven Nadler observes, “few works in the canon of Western philosophy stand at a richer intersection of intellectual traditions than Spinoza's *Ethics*” (Nadler 2014, 152). Researchers detect elements of ancient Stoicism and medieval Jewish philosophy in *Ethics* and trace how Spinoza's philosophical system departs significantly from both.^33^ Born into the Age of Reason, Spinoza engaged in critical dialogue not only with ancient Stoicism and medieval Jewish rationalism but also with contemporaries Descartes and Hobbes.^34^ While the sources and contexts of *Ethics* are traceable, the system of thought it presents remains refined, coherent, and strikingly original. Despite early suppression, as they were viewed as a challenge to organized religion, Spinoza's ideas and their influence on the European Enlightenment, German idealism, and contemporary psychology are increasingly recognized (see, for instance, Damasio 2003; Förster and Melamed 2012; Israel 2001).

### 3.3 Exploring the Commonality and Variability of the Holistic System of Traditional Chinese Medicine and the Philosophical System of Spinoza as Exemplified in *Ethics*, With Special Reference to the Role of Emotions

In retrospect, both the holistic system of traditional Chinese medicine and the philosophical system of Spinoza present themselves as coherent and original, with roots in their respective cultures. It is worth comparing and contrasting these two systems of thought.

An obvious commonality between the holistic system of traditional Chinese medicine and the philosophical system of Spinoza, as exemplified in *Ethics,* is the emphasis on the adverse effects of excessive emotions on human life. Another important commonality is the assumptions of the interconnectedness of the human body and laws of nature, along with the interconnectedness of the human body and emotions.

Nonetheless, the proposition regarding the important role of emotions in changing emotions was only a commonality on the surface. A closer examination of the two systems of thought reveals greater variability than commonality. As expounded above, Spinoza's philosophical system lays more emphasis on the role of the mind in governing excessive emotions, which involves manifold adequate ideas requiring refined reasoning and profound understanding. As for the case of traditional Chinese medicine, in its theoretical statements as well as practical illustrations of using emotions to transform emotions, there have been no explicit statements about the role of cognitions of any sort.

Both systems of thought are concerned with the well‐being of humans, attainable through understanding of and compliance with the laws of nature and the regulation of excessive emotions. In comparison, the philosophical system of Spinoza poses a higher ideal of the good life, characterized by striving for a profound understanding of the interconnectedness of nature and the human world.

Understanding through the framework and terminology of Spinoza the practice of emotion‐focused therapy in traditional Chinese medicine, we might consider its merits in constructing a scenario that changes the power of external causes, which could be conducive to powerful, adequate ideas and new emotions. Changing the environment and providing new experiences as part of the emotion‐focused therapy in traditional Chinese medicine is a dimension not touched upon by Spinoza. Strictly speaking, Spinoza's way of emotional regulation might be better characterized as a cognition‐focused one. In light of advancements in contemporary psychology, the intricate relationship between emotions, cognitions, and actions in human life is increasingly recognized (see, for instance, A. Ellis and D. J. Ellis 2014/2019; Gross 1998, 2015a, 2015b). This kind of mechanism might have operated in the illustrated examples of successful emotion‐focused therapy recorded in the *yi'an* of traditional Chinese medicine, even though the role of cognition was not explicitly reported there. In the existing historical records, we are not informed if the Chinese physicians and/or the clients were conscious of the cognitions involved before, during, and after the therapeutic process.

## 4 The Cognition‐Focused Versus Emotion‐Focused Approach to Emotional Regulation and Transformation in Contemporary Psychological Science and Psychotherapy

In evaluating the contributions and limitations of emotion‐focused therapy in traditional Chinese medicine, it is desirable to place the discussion in the context of contemporary advancements in psychological science (in general) and psychotherapy (in particular).

Spinoza's doctrine of emotions was known to Wundt, the father of psychology. Wundt further remarked that older psychology had followed the method of Spinoza and “generally offered all kinds of logical reflections about emotions” (Wundt 1896/1897, 174). In the contemporary scene, a closer examination of influential theories and practices revealed the intriguing role of cognition in the regulation and transformation of emotions.

In the field of theoretical development and research on emotion regulation, the process model proposed by Gross (1998, 2015a, 2015b) stands out as the most comprehensive paradigm. The process model of emotion regulation consists of five forms: namely, situation selection, situation modification, attentional deployment, cognitive change, and response modulation (Gross 1998). In the postulation of an extended process model (EPM), Gross (2015b) further distinguishes three stages of the emotion regulation cycle characterized by identification, selection, and implementation. Individual differences in the tendency to regulate emotions using two major strategies of cognitive reappraisal versus expressive suppression were investigated (see Gross and John 2003). The effects of cognitive control, inclusive of controlling attention and cognitive reappraisal, were further examined in functional imaging studies (see Ochsner and Gross 2005). Upon close examination, the conceptualized process of emotion regulation is essentially constituted by a cognitive approach. A further dual‐process framework of explicit and implicit emotion regulation was proposed by Gross and colleagues^35^ (Gyurak et al. 2011). It is worthy noting that even the five forms of implicit emotion regulation do not involve using emotions to change emotions, which is the essence of the emotion‐focused therapy in traditional Chinese medicine.^36^

In the past decades, the rational emotive behavior therapy (REBT) proposed and practiced by Albert Ellis and his followers is considered an influential approach in psychotherapy (see Wedding and Corsini 2014/2019). While REBT is characterized by a holistic view that highlights the interconnectedness of human thoughts, feelings, and behaviors, its therapeutic approach is basically a rational or cognitive one, as aptly defined by its chief proponents (see Ellis and Blau 1998; A. Ellis and D. J. Ellis 2011/2019). The core mechanism of REBT lies in disputing irrational beliefs/cognitions. In the presence of activating events or adversities, it is held that irrational beliefs would lead to the consequences of unhealthy negative emotions and self‐defeating behaviors. It is further postulated that a successful disputing of irrational beliefs would facilitate the emergence of effective rational beliefs/new philosophies, along with effective emotions and behavior. An REBT Self‐Help Form has been developed to facilitate the process of self‐awareness and self‐reflection. Irrational beliefs are manifold and the road to well‐being lies in one's success in disputing them (see A. Ellis and D. J. Ellis 2014/2019). Even though the interconnectedness of thoughts, feelings, and behaviors is highlighted in this influential therapeutic approach, it appears that emotions have hardly a role to play in the transformation of emotions, unlike in the case of the emotion‐focused therapy of traditional Chinese medicine.

What deserves our further review is the emotion‐focused therapy (EFT) proposed by Greenberg (2004, 2012, 2002/2015, 2010/2017). In this therapeutic approach, emotion is perceived as foundational in self‐construction and self‐organization; it is also considered essential to therapeutic change (Greenberg 2004; Pos and Greenberg 2007). With regard to the therapeutic process, six principles of emotion changes have been formulated and expounded: namely, awareness, expression, regulation, reflection, changing emotion with emotion, and corrective emotional experience (Greenberg 2012, 2010/2017). In particular, the role of the therapist in coaching clients to work through their feelings is highlighted (Elliott et al. 2004; Greenberg 2002/2015; Greenberg and Goldman 2019). Case formulation (a working hypothesis about the client's core painful emotion) constitutes an important initial step, which is followed by the therapist's marker‐guided interventions on the moment‐to‐moment experience of a client in session, with “marker” referring to the underlying emotional processing difficulties of a client (Greenberg 2002/2015; Greenberg et al. 1993; Greenberg and Paivio 1997/2003). In therapeutic practice, with the empathetic assistance of the therapist, special emphasis is given to uncovering and leaving the maladaptive primary emotions (Greenberg 2002/2015; Leung Ho 2021). For accessing new emotions, numerous methods have been proposed, such as shifting attention, making positive imagery, using psychodrama to activate emotion, remembering another emotion, and cognitively creating a new meaning (Greenberg 2004, 2002/2015).

A closer examination of the mechanism of EFT reveals that cognition also plays an indispensable role in the process of emotional awareness, regulation, reflection, and transformation. Dialogues between the therapist and the client are important for attaining psychotherapeutic change. The application of two‐chair work and empty‐chair work also essentially involves the client's self‐dialogue. In characterizing emotional change in EFT as a dialectical constructivist perspective, the role of language in processing the aroused emotions and in creating new meanings in the therapeutic sessions is recognized (Greenberg 2012; Greenberg and Pascual‐Leone 2001). It is worth noting that Greenberg (2012) has explicitly lent support to a psychotherapy integration moving toward a dynamic, emotion‐focused, and cognitive behavioral approach, wherein emotion is recognized as central to therapeutic change. In highlighting the importance of emotion schemes in human functioning, Greenberg (2012) has perceived them as a set of highly integrated affective‐cognitive units.

Greenberg (2004) echoes Spinoza's insight that emotions are needed to change emotions. More specifically, it is held that emotional change cannot be achieved by a rational process of understanding or explanation but rather by the generation of a new emotional response (Pos and Greenberg 2007). “Changing emotion with emotion” is one of the six principles in emotional change, and the one which Greenberg perceived as “probably the most important way of dealing with emotion in a therapy” (Greenberg 2012, 704). Specifically, this principle refers to the mechanism of undoing primary maladaptive emotions (e.g., fear and shame) by activating other, more adaptive emotions such as assertive anger, the sadness of grief, and compassion for self (Greenberg 2012, 2002/2015, 2010/2017). It is necessary to comprehend this mechanism in the context of previous phases of the therapeutic process, which facilitate and make possible this kind of transformation.

It is worth noting that “using emotion to change emotion” is one of the six principles of EFT, as well as constituting the core principle of emotion‐focused therapy in traditional Chinese medicine. The similarity between the two therapeutic approaches lies in their full recognition of the transformative power of emotions as well as their emphasis on working with emotions. The methods applied are nonetheless different. EFT is characterized by a series of systematic and coherent therapeutic sessions that place value on the relationship between the therapist and the client, during which adequate therapeutic skills are applied through diverse types of dialogues, including two‐chair work and empty‐chair work (see Elliott et al. 2004; Pos and Greenberg 2007). By contrast, the method applied by master physicians in traditional Chinese medicine is characterized by the shrewd design of a specific scenario in one's living environment, often with the collaboration of a person who is close to the client. Such a method of rendering an embodied alternate experience for clients in the real world could undo a client's excessive and obsessive emotion by generating a new emotion that is strong and contrasting.

## 5 The Contributions and Limitations of Emotion‐Focused Therapy in Traditional Chinese Medicine

Picturing contemporary psychological science and psychotherapy as the “ground,” the “figure” of emotion‑focused therapy in traditional Chinese medicine is well positioned to reveal its distinctive characteristics and potential contributions.

As discussed earlier in Section 2, the classical texts of traditional Chinese medicine, alongside case records across the Jin, Yuan, Ming, and Qing dynasties, affirm the power of one emotion to transform another. In practice, physicians identified a maladaptive, obsessive core emotion and then designed an embodied alternate experience to elicit a contrasting emotion. These designs ranged from simple to complex, depending on the client's condition and environment. When compared with the procedures of contemporary psychotherapy, the potential strengths and contributions of this traditional approach stand out. Essentially, it demonstrates that rapid emotional transformation can occur outside formal therapy, often in naturalistic settings such as the home. Its effectiveness depends on the physician's creative design of the scenario, implemented through a trustful collaboration between the physician and a family member of the client, or with the client directly. In this respect, emotion‑focused therapy offers a valuable supplementary pathway for clients in a pathological mood state who are unable or unwilling to engage in structured therapeutic sessions.

Evaluated against the advancement of contemporary psychotherapy, the limitations of the emotion‐focused therapy of traditional Chinese medicine are evident. In the first place, debriefing about the rationale of setting up the unique scenario to elicit a new, intensive emotion was not explicitly reported in the case records. We also do not know if there was any integration of the clients’ self‐awareness and self‐reflection in the therapeutic process subsequent to the dramatic emotional transformation. Even though the original, obsessive emotion was removed, the client might not have understood what mechanisms were involved. If a client does not understand the interrelations of their own thoughts, emotions, and behaviors through self‐awareness and self‐reflection, they will not be in the position to cope with the next challenge of excessive emotion or to maintain their well‐being.

It is worth noting that reports and positive evaluations of emotion‑focused therapy in traditional Chinese medicine live on in contemporary literature (see, for instance, Tao and Gao 2012; M. Wang 1991; B. Zhang 1996). However, we do not witness the collection of newly designed therapeutic cases using this approach. This might be attributed to the differentiation of roles between physicians and therapists in present‐day China. In revitalizing this wisdom, it is important to safeguard new practices against the mere copying of past designs. In the case of any temporary deception being involved, ethical principles should be fully deliberated. Looking forward, we need to revive the insight of this approach and to develop new designs by drawing on its principles. Specifically, we can embrace the insight of affirming the need for a strong emotion to overcome a core, obsessive emotion. We can further uphold two principles that have made this emotion‐focused therapy work well in the past: (1) With shrewdness and creativity, it is essential to design a unique scenario that provides an embodied alternate experience for the clients in the real world, one conducive to a new, intensive emotion; (2) To implement the therapy, it is crucial to build in a naturalistic setting a trustful collaboration between the therapist and a person having a close relationship to the client, if the client does not already have complete trust in the therapist. Recognizing the merits of contemporary psychotherapy in Western culture, we can further integrate a third principle: (3) To consolidate the effects of the emotion‐focused therapy, it is best to conduct a debriefing about the unique situation designed for emotion transformation. This should be followed by an intensive cultivation of self‐awareness and self‐reflection in the client, with a view to attaining a deeper understanding of the interrelatedness of the environment and self on the one hand, and a more refined discernment of the interconnectedness of the human body, cognitions, emotions, and actions on the other.

## 6 Concluding Thoughts

Emotion‐focused therapy (EFT) in contemporary psychology cites Spinoza as the precursor of the idea of “using emotion to change emotion,” which constitutes one of the six principles of this therapeutic practice (Greenberg 2004, 2012, 2010/2017). Nonetheless, there is no further elaboration of any ideas from Spinoza's system of thought in the vast literature concerning this practice, nor are there any traces in the practical application of Spinoza's refined, rational approach in overcoming passion. In the field of cognitive‐focused therapy, Albert Ellis—the founder of rational emotive therapy (RET), later known as REBT—has explicitly attributed the foundation of RET to the Stoic viewpoint, highlighting the way in which people are disturbed not by things but by their view of things (Ellis and Dryden 1987).^37^ Numerous philosophical and psychological influences on the formulation and development of REBT have been further named by Ellis, with the writing of Spinoza being one of them (Ellis and Dryden 1987; A. Ellis and D. J. Ellis 2014/2019).^38^ In retrospect, there is only limited actual influence of Spinoza's system of thought evident in the shaping of contemporary psychotherapies,^39^ despite the richness and sophistication of Spinoza's ideas relevant to therapy and development.

Essentially, Spinoza (1677/2018) portrays the body, emotions, and ideas as an integrated whole. The microdynamics of mind that involve active versus passive emotions, adequate versus inadequate ideas, and the interpretation of external causes, conceptualized by Spinoza, are subtle and inspiring. His postulation of the three kinds or modes of cognition is exceptionally refined, illustrating a continuous, active process in our rational understanding of the essence of reality, which is conducive to overcoming passive emotions and attaining a good life. Understandably, applying Spinoza's philosophy in self‐therapy requires a high capacity for introspection and reflection. It can nonetheless serve as a rich resource for exploration and creative adaptation within contemporary psychotherapies. Furthermore, Spinoza's system of thought is in the position to contribute to emotion‐focused therapy in traditional Chinese medicine in two significant ways. First, its holistic view of body, emotions, and ideas can inspire the design of new scenarios for embodied experiences that elicit a strong alternative emotion. Second, its rationalist emphasis on clarifying and accumulating adequate ideas in relation to external causes can benefit clients during debriefing, subsequent to a dramatic, emotion‐based intervention. Refined in these ways, emotion‐focused therapy practiced in traditional Chinese medicine may be revitalized as a promising therapeutic approach.

In characterizing the structure and unfolding content of this article, it is gratifying to use the metaphor of a symphony, in which there is a key musical theme or idea present in the developing movements of the musical piece, constituting a coherent whole. The major theme of this symphony is reflected in the article title: “Emotion‐Focused Therapy: A Perspective From Traditional Chinese Medicine.” Introducing the origin and key features of emotion‐focused therapy in traditional Chinese medicine sets the stage in the first movement. The second movement proceeds into a deeper elaboration of and dialogue between the two profound thought systems (traditional Chinese medicine and Spinoza's philosophy), where a similar insight emerges regarding the necessity of a contrasting, strong emotion in transforming another excessive emotion. The variability observed between these two holistic systems seems to be more pronounced than their commonality. The third movement, covering key attainments in contemporary psychological science and psychotherapy, leads the way to the crescendo in the fourth movement: an evaluation of the contributions and limitations of emotion‐focused therapy in traditional Chinese medicine in light of contemporary psychotherapeutic advances. The juxtaposition of contrasting ideas in different temporal and spatial settings is illuminating here. Readers are invited to engage in listening to the subtleties of this musical piece and its lingering sound. An attempt to “perform” the symphony is also encouraged, which might mean a fresh, meaningful interpretation of its theme and movements.

## Conflicts of Interest

The author declares no conflict of interest.

## Data Availability

Data sharing not applicable to this article as no datasets were generated or analyzed during the current study.

## Endnotes

1 See, for instance, the chapters “陰陽應象大論” [“On the Phenomena of Yin‐Yang”] and “調經論” [“On Modulation of Qi Flow in the Conduits”], compiled in 黃帝內經素問校释 [*Annotations on Basic Questions of The Yellow Emperor's Inner Canon*] (1982/1995), vol. 1, 62–97 & vol. 2, 766–794, respectively. See also “本神” [“Acupuncture Based on the State of the Mind”] in 黄帝内经灵枢三家注 [*Divine Pivot of The Yellow Emperor's Inner Canon: Three Annotation Traditions*] (Y. Wang 2013), vol. 1, 123–133.

2 These phrases, each of which was expressed in a separate paragraph, were introduced in “陰陽應象大論” [“On the Phenomena of Yin‐Yang”] and “五運行大論” [“Patterns of Climatic Changes and Outbreak of Disease”] in *Basic Questions*.

3 How the excess of emotion can harm the *shen* 神 (spirit) and the *qi* 氣 (life energy) is also emphasized in *The Yellow Emperor's Inner Canon*. On the impact of excess emotion on the spirit, see “Acupuncture Based on the State of the Mind,” in *Divine Pivot* of *The Yellow Emperor's Inner Canon: Three Annotation Traditions* (Y. Wang 2013), vol. 1, 123–133. On the impact of excess emotion on life energy, see “On the Phenomena of Yin‐Yang,” “On Pain,” and “On Modulation of Qi Flow in the Conduits,” in *Annotations on Basic Questions of The Yellow Emperor's Inner Canon* (1982/1995), vol. 1, 62–97, 497–509, and vol. 2, 766–794, respectively.

4 Two large‐scale collected works, published in the Ming dynasty and the Qing dynasty, respectively, are recognized as important references: Jiang, Quan 江瓘 (1505–1565/2014), 名醫類案 [*Classified Case Records of Master Physicians*], and Wei, Zhixiu 魏之琇 (1722–1772/1997), 續名醫類案 [*A Further Edition on the Classified Case Records of Master Physicians*]. Whereas the original edition of *Classified Case Records of Master Physicians* contains more than 2300 cases, the later edition from the Qing dynasty contains nearly 5000 cases. A similar reference book compiled by Yu Zhen 俞震, which contains more than 1,500 cases, is generally perceived as a more refined collection with insightful commentaries; see Yu Zhen (1709–1799/2008), 古今医案按 [*Remarks on Traditional and Contemporary Yi'an*].

5 It is worth mentioning in this context that an ethnographic study of the clinical practice of Chinese medicine did not observe or report any application of using one emotion to overcome another emotion. The method observed in the clinical practice was characterized as *tiao* 調 (attuning), which means helping the patient to adjust through multiple dimensions, including herbal treatment and persuasion to transform emotions and change perspectives (Y. Zhang 2007).

6 Originally published in 景岳全書 [*The Complete Works of Jingyue*], cited by Zhang Bohua 张伯华, 中医心理学 [*Chinese Medical Psychology*], 184–185.

7 Originally published in 洄溪醫案 [*The Therapeutic Case Records of Huixi*], cited by Zhang Bohua, *Chinese Medical Psychology*, 189.

8 Zhang Zihe (1156–1228/2017), *Confucians’ Duties to Parents*, 261–262.

9 Joy has been regarded as an emotion that can overcome anger, deliberation, and sadness, in addition to worry. See the *yi'an* collected in Wang Maohe 汪茂和 (Ed.) (1991), 中国养生宝典 [*Treasured Classics in Chinese Yangsheng*], 358–362, 366–367.

10 See Yu Zhen (1709–1799/2008), *Remarks on Traditional and Contemporary Yi'an*, 170.

11 Zhang Zihe (1156–1228/2017), *Confucians’ Duties to Parents*, 261–262.

12 See *Annotations on Basic Questions of The Yellow Emperor's Inner Cano*n (1982/1995), vol. 1, 62–97 (“On the Phenomena of Yin‐Yang”) & vol. 2, 860–890 (“Patterns of Climatic Changes and Outbreak of Disease”).

13 See, for instance, “Acupuncture Based on the State of the Mind,” in *Divine Pivot of The Yellow Emperor's Inner Canon*, vol. 1, 123–133.

14 The extensive corpus of ancient Chinese scripts—including Oracle Bone Script, Bronze Script, Great Seal Script, and Small Seal Script—enables specialized etymological and semantic analyses of early Chinese words. It is worth noting that monosyllabic words predominated in Old Chinese; see, for instance, Wang Li 王力 (1962/1997), *Gudai Hanyu* 古代漢語 [*Old Chinese*]. For an elaborate discussion of etymological and semantic analyses of emotion words in Chinese culture, see W.‐c. Wong (2025). Constructing a Genealogy of Emotions (*Qing* 情) in Chinese Culture Through the Lens of Etymological and Semantic Analyses. *Monumenta Serica. Journal of Oriental Studies*, *73*(2), 309–341.

15 The two monosyllabic “emotion words” (i.e., *xi* 喜 and *le* 樂) and one monosyllabic “emotion‐laden word” (i.e., *xin* 心), as recorded in the Oracle Bone Script, appear in *Kōkotsu kinbun jiten* 甲骨金文辭典, a meticulously researched and illustrated lexicon compiled by Mizukami Shizuo 水上靜夫. Based on the gestalt of the ancient script forms, Mizukami Shizuo argued that their original meanings were not related to emotions. The ancient scripts of *xi* likely referred to the eating of well‐boiled food. *Le* referred to a type of plant in the Oracle Bone Script and a musical instrument in the Bronze Script. The gestalt of *xin* in ancient script forms appears to depict an animal heart. See *Kōkotsu kinbun jiten* (1995), vol. 1, 236–237, 494–495, 684–685.

16 Most emotion words created in this way employed a method that integrated a phonological component with the orthographic and semantic elements of *xin*. See *Kōkotsu kinbun jit*en (1995), vol. 1, 494–530; *Hanyu duogongneng ziku* 漢語多功能字庫 (*Multi‐Function Chinese Character Database*) (2014), the section on *xin* as the principal component.

17 See *Kōkotsu kinbun jiten*, vol. 1, 494–530; *Multi‐Function Chinese Character Database*, the section on *xin* as the principal component. Where there are differences in tracing the earliest script of a certain emotion or emotion‐laden word in the above two tools, *Kōkotsu kinbun jiten* is a more reliable source.

18 See *Kōkotsu kinbun jiten*, vol. 1, 494–530; *Multi‐Function Chinese Character Database*, the section on *xin* as the principal component; *Zhengzhong xingyinyi zonghe dazidian* 正中形音義綜合大字典 (S. Gao 1971/1998), 483–533.

19 See *Liushu gu* 六書故 (Southern Song dynasty/2012), vol. 1, 13:9; *Zhengzhong xingyinyi zonghe dazidian* (S. Gao 1971/1998), 504. In a bamboo strip text titled “Xing zi ming chu” 性自命岀 (“Human nature is brought forth by decree”), unearthed in 1993 in a tomb at Guodian 郭店, *qing* is explicated as the manifestation of human nature (“*qing sheng yu xing*” 情生於性), while human nature is described as originating from the mandate of nature. This Confucian classic can be traced back to approximately 300 BCE. See Li Ling 李零 (2002), *Guodian Chujian jiaoduji* 郭店楚簡校讀記 [*Annotations on the Guodian Chu Strips*], 105‐120; Meyer (2012), *Philosophy on Bamboo*. *Text and the Production of Meaning in Early China*, 7 & 131–174.

20 It appears that the concept of *dao* in Daoism has its roots in the *Yijing*. The naïve dialectic underlying the *Yijing* also seems to have inspired the dialectical thinking expressed in the *Laozi*. Core ideas in Confucianism—such as self‐cultivation and the interaction between *tian* 天 (heaven) and *ren* 人 (person)—likewise have their origins in the *Yijing*.

21 See Huang Shouqi 黄寿祺 and Zhang Shanwen 张善文 (2002), *Zhouyi yizhu* 周易译注.

22 The legend of Fuxi is recounted in Part II of the Great Treatise (Xici, xia 繫辭下) of the *Yizhuan* 易傳. See *Zhouyi dazhuan jinzhu* 周易大傳今注, annotated by Gao Heng 高亨 (2010). The primary forces of yin and yang were also highlighted in the Great Treatise; see Gao Heng (2010).

23 See Yu Dunkang 余敦康 (2005), *Yixue jinxi* 易學今昔. The construction of the Sixty‐Four Hexagrams is generally attributed to Fuxi, though some sources also credit Shennong 神農 or King Wen 文王, the founder of the Zhou dynasty; see Huang Shouqi and Zhang Shanwen (2002).

24 The judgment or explanatory text associated with each hexagram, known as *Tuan* 彖, is credited to King Wen. The line statements (*Yaoci* 爻辭) are attributed to Duke Zhou, the son of King Wen. See Kong Yingda 孔颖达 (574–648/1999), *Zhouyi zhengyi* 周易正义 [*Setting Right the Meanings of the Zhouyi*], *juan* 1, 9.

25 The first round of interpretations regarding the images and texts of the *Yijing* emerged around the Spring and Autumn period (770‐476 BCE), the Warring States period (475–221 BCE), and the Western Han dynasty commencing in 206 BCE; see Huang Shouqi and Zhang Shanwen (2002). Among these interpretations—collectively known as the *Yizhuan*  (also referred to as the “Ten Wings”)—the two‐part Great Treatise has generally been designated a philosophical text; see Zhu Bokun 朱伯崑, Li Shen 李申, and Wang Deyou 王德有 (Eds.) (1993/2004), *Zhouyi zhishi tonglan* 周易知识通览 (*An Encyclopedia* on *Zhouyi*).

26 In Part II of the Great Treatise, this portrayal appears in the form of a rhetorical question:  *Zuoyizhe, qi you youhuan hu*? 作易者, 其有憂患乎? (“When the authors composed the *Yijing*, was it not driven by their crisis‐consciousness and worry?”) Notably, the use of this classical Chinese rhetorical pattern implies an emphatic yes in answer; see Gao Heng (2010), 434.

27 The continuous annotation and discussion of the *Yijing* and the *Yizhuan* throughout Chinese intellectual history is remarkable. Despite substantial refinements over time, the core message—emphasizing the importance of understanding subtle, interactive changes in nature and the human world, and responding appropriately—has remained a consistent point of consensus. See, for instance, Lin Zhongjun 林忠军 (2005), *Zhouyi Zhengshixue chanwei* 周易郑氏学阐微; Wang Bi 王弼 (227–249/1999), *Zhouyi zhu* 周易注; Kong Yingda 孔颖达 (574–648/1999), *Yijing zhengyi* 周易正义; Zhu Xi 朱熹 (1130–1200/1997), *Yi benyi* 易本義; Wang Fuzhi 王夫之 (1617–1692/2011), *Zhouyi neizhuan* 周易內傳; Li Guangdi 李光地 (Comp.) (Qing dynasty/2006), *Zhouyi zhezhong* 周易折中.

28 In the impactful Confucian classic of “Zhongyong” 中庸 (“The Doctrine of the Mean”), Zisi 子思 expressed the following: “喜怒哀樂之未發為之中。發而皆中節謂之和。” (While there are no stirrings of pleasure, anger, sorrow, or joy, the mind may be said to be in the state of Equilibrium. When those feelings have been stirred, and they act in their due degree, there ensues what may be called the state of Harmony.) See *Shisanjing guzhu* 十三經古注 (vol. 5: *Liji* 禮記), 2014, 193. English translation by Legge 1815–1897; see https://ctext.org/liji/zhong‐yong.

29 See Confucius 孔子 (ca. 551–479 BCE/2006). “Xianwen” 憲問. In *Lunyu xin jiaoshi* 论语新校释 [*The New Annotations of the Analects*], 357.

30 The *Liji*, which incorporates classical Confucian texts, was compiled in the Western Han dynasty. In the chapter “Liyun” 禮運 of the *Liji*, there is a scheme classifying *qíng* (情) into seven kinds: *xi* 喜 (pleasure), *nu* 怒 (anger), *ai* 哀 (grief), *ju* 懼 (dread), *ai* 愛 (affection), *wu* 惡 (disgust), and *yu* 欲 (desire). The text further states that a sage would regulate these seven emotions of the people with rites. Notably, this scheme both converges with and diverges from the seven kinds of emotion highlighted in traditional Chinese medicine. See *Shisanjing guzhu* (vol. 5: *Liji*), annotated by Zheng Xuan (127–200) et al. 2014, 964. In the chapter “Yueji” 樂記 of the *Liji*, the arrangement of rituals and music by ancient emperors is explained as a means of regulating people's desires. It is further stated that “great music resonates in harmony with Heaven‐and‐Earth; great rites share the regulating principles with Heaven‐and‐Earth.” (The original Chinese text reads as follows: “大樂與天地同和, 大禮與天地同節”). See *Shisanjing guzhu* (vol. 5: *Liji*), 1015. For Xunzi's view on the regulation and cultivation of emotions, see his essays “Lilun pian” 禮論篇 (“On Rituals”) and “Yuelun pian” 樂論篇 (“On Music”). See *Xunzi jijie* 荀子集解 1998, vol. 2, 346–378 (“On Rituals”) and 1998, 379–385 (“On Music”).

31 The term “affectus,” originating from Latin, is often translated into English as “emotion” or “affect.” For example, in the first completed translation of *Ethics* by George Eliot (Spinoza 1677/2020) and its contemporary translation by Silverthorne and Kisner (Spinoza 1677/2018), “affectus” is translated as “emotion.” In contrast, LeBuffe (2009) translates Spinoza's use of “affectus” as “affect” in his book chapter, “The Anatomy of the Passions.” The choice of translation requires careful consideration of Spinoza's original meaning of “affectus,” along with the contemporary meanings of “emotion” and “affect” in philosophical and psychological contexts. First, it is important to recognize the intriguing relations between the body, emotions, and ideas in Spinoza's original application of “affectus.” It is also worth mentioning that “affectus” is translated as “Affekt” in the German version of *Ethics* (e.g., Spinoza 1677/1989), whereas Wundt (1896/1897, 1896/2022), the father of psychology, endorses the translation of “Affekt” as “emotion” by Judd in the English version of his work, *Outline of Psychology*. According to Wundt, an emotion is a unitary whole of interconnected affective processes that has a “temporal course,” which “exercises a more intense present and subsequent effect on the interconnection of psychical processes” (Wundt 1896/1897, 170). In a contemporary setting, Plamper considers “emotion” to be the best choice of word in academic discourse because it “includes dimensions of appraisal, signification, object‐directedness and consciousness” (Plamper 2015, 455). In the *APA Dictionary of Psychology* (2007/2018), emotion is defined as “a complex reaction pattern, involving experiential, behavioral, and physiological elements” (see https://dictionary.apa.org/emotion). On the other hand, “affect” is generally considered as a construct that incorporates emotions, moods, temperament, and traits (see, for instance, Davidson 1994; Ekman 1994). In the emerging field of affective sciences, “affect” is a broad term often referring to “mental states that include emotions, moods, attitudes, interpersonal stances, and affect dispositions” (Frijda and Scherer 2009, 10). The genesis and changes in meaning regarding the terms “affectus,” “Affekt,” “emotion,” and “affect” throughout history constitute a complex phenomenon, which makes the creation of a satisfactory translation a highly difficult task. Taking into consideration the original meaning of Spinoza's use of “affectus” in *Ethics* and the academic discourses in contemporary settings, “emotion” is proposed as a more appropriate English translation of “affectus.” Greenberg also uses “emotion” as the English translation, while attributing Spinoza as a pioneer in regard to sharing the insight of the necessity of a strong emotion to change emotions (Greenberg 2004). It is also worth noting that any translation of the Latin word “affectus” used by Spinoza into a modern English word would unavoidably lose the nuances of its original meaning. It is thus necessary to delve into Spinoza's philosophical system in *Ethics* to understand the meaning of “affectus.”

32 God is defined by Spinoza as the causes and laws of Nature, and as the only substance that presents in all entities, which are modes of the substance (see Part I of *Ethics*, Spinoza 1677/2018).

33 For observations of Stoic elements in *Ethics* and its departure from Greek and Roman Stoicism, see, for instance, Peteboom (1994/2001), “Stoic Psychotherapy in Descartes and Spinoza”; Nadler (2006), *Spinoza's Ethics: An Introduction*; Lloyd (2024), *Reading Spinoza in the Anthropocene*. For areas of convergence and divergence between medieval Jewish philosophy and Spinoza's system of thought, see, for instance, Harvey (1981/2001), “A Portrait of Spinoza as a Maimonidean”; Nadler (2014), “Virtue, Reason, and Moral Luck: Maimonides, Gersonides, Spinoza.”

34 Biographical studies on Spinoza reveal that he received formal Jewish education in the Portuguese community of Amsterdam, followed by private Latin instruction under Francis van den Enden, an ex‐Jesuit. He is also reported to have studied at the University of Leiden. For details, see Nadler (1999), *Spinoza: A Life*; Curley (1994), “Introduction” in *A Spinoza Reader. The Ethics and Other Works*. For a detailed study on Spinoza's critical dialogue with Descartes, see Jaquet (2018), *Affects, Actions and Passions in Spinoza: The Unity of Body and Mind*. For a perceptive discussion of how Spinoza transforms Hobbesian ideas, see LeBuffe (2021), “Spinoza and Hobbes.”

35 Whereas explicit emotion regulation requires conscious effort that involves monitoring and demands some level of insight and awareness, implicit emotion regulation is evoked automatically and can take place in the absence of insight and awareness and be completed without monitoring (Gyurak et al. 2011, 401).

36 The five forms of implicit emotion regulation are (1) emotional conflict adaptation, (2) habitual emotion regulation, (3) emotion regulatory goals and values, (4) emotion regulation as a result of affect labeling, and (5) error‐related regulation (Gyurak et al. 2011, 404–407).

37 Ellis reported that he became increasingly dissatisfied with psychoanalysis as a form of treatment and began experimenting with different therapeutic forms in the early 1950s. He stated that, during this period, the works of Greek and Roman Stoic philosophers, including Epictetus and Marcus Aurelius, had a major influence on his thought (see Ellis and Dryden 1987).

38 Apart from highlighting Stoicism as a major philosophical influence on RET, Ellis also mentioned the influence of Kant, Spinoza, and Schopenhauer in regard to their writings on the power (and limitations) of cognition and ideation. He further acknowledged that RET owes a debt to philosophers of science such as Popper, Reichenbach, and Russell, alongside existential thinkers, including Heidegger and Tillich. With respect to major psychological influences, Ellis credited the works of Horney and Adler (see Ellis and Dryden 1987, for a discussion of these philosophical and psychological influences).

39 There have been some discussions of the similarities between the thought system of Spinoza and the system of psychoanalysis (see, for instance, Bernard 1946, 1977; Neu 1977). Nonetheless, there is no evidence showing any direct, substantial influence of Spinoza's ideas on the development of theory and practice in psychoanalysis. In this context, Freud's remarks in a letter to Bickel about his silence on Spinoza's ideas, dated 1931 and rediscovered in 1974, are thought‐provoking (Hessing 1977, 227): “My dependence on the teachings of Spinoza I do admit most willingly. I had no reason to mention his name directly, as I got my presumptions not from studying him, but from the atmosphere he created. And as such, any philosophical legitimation was of no great importance to me at all.” For further details, see the German and translated English texts of the letter attached to Hessing's (1977) article, 227–228. Notably, there has been a recent call to integrate Spinoza's philosophy into contemporary therapeutic practice in psychoanalysis (Miller 2022). Regarding contemporary psychotherapeutic practices categorized under the influential stream of cognitive behavioral therapy (CBT), there is no evidence of a legacy of Spinoza's philosophy, although CBT is often credited with drawing on Stoicism (see Robertson 2010; Leahy and Martell 2021).
